# Multifacet of Cornea Patch Graft in Anterior Segment Diseases

**DOI:** 10.1155/2019/6862487

**Published:** 2019-11-11

**Authors:** Prema Chendran, Yong Meng Hsien, Wan Haslina Wan Abdul Halim

**Affiliations:** Department of Ophthalmology, University Kebangsaan Malaysia Medical Centre (UKMMC), Jalan Yaacob Latif, 56 000 Bandar Tun Razak, Kuala Lumpur, Malaysia

## Abstract

Incidence of cornea melting or perforation is commonly seen in variety of cornea conditions. It can cause debilitating vision loss and impair patient's daily activity. Several techniques have been described to surgically treat cornea perforation and melting. In this article, a series of corneal diseases treated with corneal patch graft are highlighted to relate different approach of cornea patch graft surgery. Post-operative management and complications are discussed.

## 1. Introduction

Cornea patch graft has been advocated as the surgical procedure to treat variety of anterior segment pathologies. It involves patching the affected area with full thickness or partial thickness corneal donor tissue. The aim is to restore globe integrity and prevent further inflammation that could lead to devastating complications such as endophthalmitis. Cornea perforation and melting can occur in infectious keratitis, autoimmune diseases such as rheumatoid arthritis, ocular trauma and ocular surface disorder.

Patch graft materials are derived from cornea, sclera, pericardium and dura mater [1]. Scleral patch graft is commonly used in anterior segment pathologies because it is relatively cost effective and easy to preserve. However due to its avascular property it is often associated with progressive tissue necrosis or melting [2]. Pericardial graft is used as patch graft in glaucoma drainage devise (GDD) exposure prevention. It is commercially available, no need to be dependent on eye bank for its availability and has high sterility but has tendency to develop graft thinning and subsequent exposure of the GDD [3] besides being expensive.

Cornea patch graft has been indicated in cases of corneal perforation, cornea thinning [4], scleral thinning or as prophylaxis to prevent the exposure of GDD [1]. The advantages of cornea patch graft compared to others is that: it is translucent, less chances of graft melting and conjunctiva retraction [1]. It can provide good tectonic support for the ocular wall as the tissue has good strength and rigidity [1]. This case series of corneal diseases treated with corneal patch graft in UKMMC is presented. The author discuss about the post-operative management that was tailored individually to each patient.

## 2. Case History

### 2.1. Case 1

**Figure 1:**
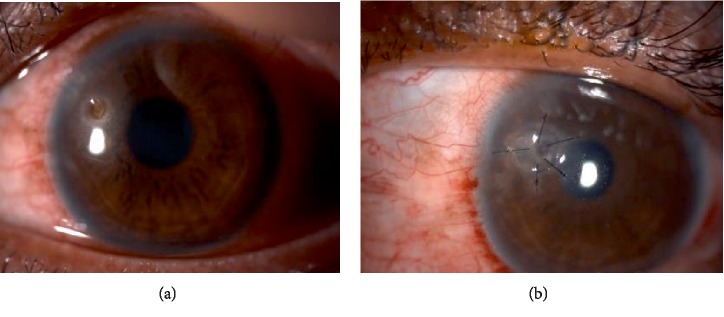
(a) Presence of stromal ring infiltrate with central cornea perforation and shallow anterior chamber. (b) Cornea patch graft applied. Post-surgery 4 months.

A 32-year-old gentleman, presented to eye casualty with sudden onset left eye redness and discomfort. He had history of foreign body entering into his left eye while hammering 1 week prior to that. Examination revealed deeply seated rust ring on the anterior cornea stroma. Failure to remove the rust ring completely resulted in corneal ulceration 1 week later. Topical antibiotics and lubricants were initiated. However, he started to develop cornea thinning. Despite being treated with intensive antibiotics and lubricants, the cornea thinning progressed to cornea perforation with flat anterior chamber (AC) (Figure 1(a)). He underwent series of nonsurgical (cornea glue, bandage contact lens (BCL) application) and surgical (AC reformation and cornea patch graft) intervention. He was able to regain his vision to 6/9 with presence of stable cornea patch graft during last review (Figure 1(b)).

### 2.2. Case 2

**Figure 2:**
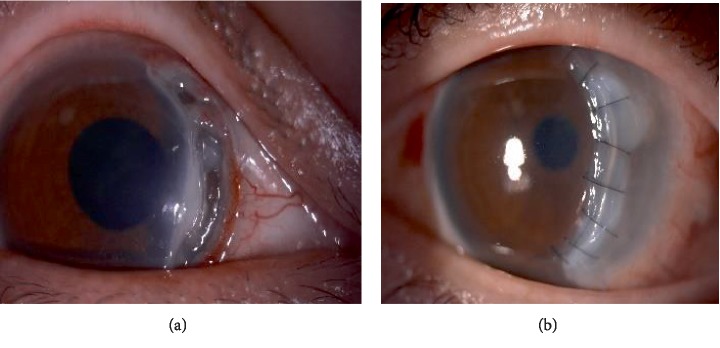
(a) Peripheral cornea thinning from12 to 5 o'clock. (b) Post banana shaped corneal patch graft.

A 38-year-old gentleman with underlying diabetes mellitus (DM) type I, hypertension, end stage renal failure (ESRF), right eye pseudophakia presented with right eye blurring of vision associated with photophobia and watery eye for 1 month. Examination revealed right eye vision of 6/60 pinhole 6/36. He had a furrow cornea thinning from 12 to 5 o'clock with overhanging edge where there's only Descemet remaining. There's adjacent cornea epithelial defect but no cornea perforation seen (Figure 2(a)).

Systemic examination and blood investigations were normal. He was diagnosed having Mooren's ulcer. Topical corticosteroid and antibiotic, systemic immunosuppressant and collagenase inhibitor were commenced. He underwent conjunctival resection and cryotherapy twice and amniotic membrane patch graft once before a banana shaped corneal lamellar patch graft was performed (Figure 2(b)). His vision post operatively at 4 months improved to 6/18 pinhole 6/12. Post operatively he developed secondary open angle glaucoma secondary to chronic steroid usage and required topical anti-glaucoma.

### 2.3. Case 3

A 46-year-old Malay gentleman, without any comorbid, presented with progressively worsening left eye vision associated with pain and redness for 1 year duration. Symptoms started to appear 6 months post pterygium excision. His left eye vision on hand motion only. On examination, there was a crescent shape peripheral corneal thinning from 4 to 1 o'clock. The remaining cornea with its overhanging edge remains oedematous. Examination of fellow eye was normal.

**Figure 3:**
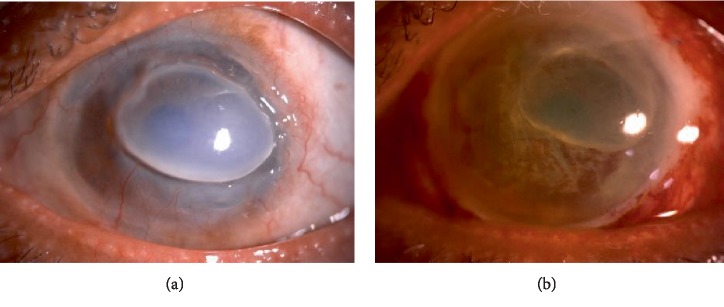
(a) Showing progression of cornea melt to 360 degrees. (b) Whole cornea patch graft.

He was investigated for underlying autoimmune or related diseases. A diagnosis of Mooren's ulcer were made and he was started on systemic and topical immunosuppression therapy. Despite this treatment, 3 months later, his eyes showed disease progression where there was continuous cornea melting to almost 360 degree (Figure 3(a)) for which he underwent conjunctival resection. However, the cornea keratolysis worsened and he was subjected to tectonic keratoplasty (cornea scleral patch graft) (Figure 3(b)). His cornea showed signs of activity stabilization 4 months post operatively.

### 2.4. Case 4

A 42-year old gentleman with underlying DM, hypertension with history of Steven Johnson Syndrome (SJS) 10 years ago presented with left progressive blurring of vision with occasional eye redness and discomfort. He had history of bilateral myopic laser refractory surgery done 3 years prior to this.

**Figure 4:**
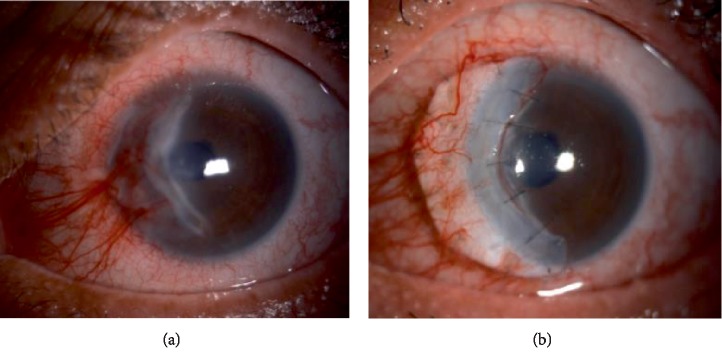
(a) Peripheral cornea melting on left eye. (b) Post banana shaped corneal patch graft on left eye.

Examination revealed right eye vision 6/24 and left eye counting finger. Both eyes showed presence of extensive peripheral cornea thinning. Peripheral edge of left cornea had slow leak but with deep anterior chamber (Figure 4(a)). Other examinations were unremarkable.

Series of investigation were taken to rule out autoimmune related diseases. He was diagnosed as Mooren's ulcer. He was started on systemic immunosuppressant, collagenase inhibitor and topical corticosteroid and antibiotic. He was scheduled for left eye cornea banana-shaped patch graft (Figure 4(b)). Post operatively his vision improved to 6/18 at 3 months and it remains stable without any recurrence.

### 2.5. Case 5

A 32-year-old Myanmar lady, complained of right eye discomfort with recurrent eye redness for the past 2 years. The eye redness usually resolve temporarily with over the counter eye drop. She noticed the presence of bluish discoloration at the right nasal conjunctiva for the past 1 year which was increasing in size. Her vision on both eyes were 6/6. She had history of trauma to right eye during childhood.

**Figure 5:**
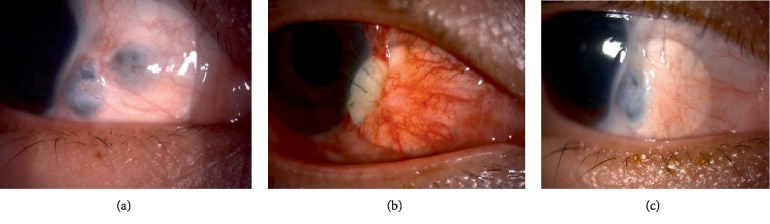
(a) Presence 2 area of scleral thinning at inferonasal quadrant. (b) Immediate post-operative scleral patch graft extending up to inferonasal limbal area. (c) Post-operative 5 months once the scleral melting has stabilized.

Right eye examination showed two locations of scleral thinning with visible uveal tissue at inferonasal quadrant with adjacent cornea scarring (Figure 5(a)). Other ocular examination were normal. Her infectious and autoimmune disease screening were negative. She was given topical lubricants and topical antiglaucoma (prophylaxis).

She underwent cornea patch graft for the scleral thinning (Figure 5(b)). Post-operatively she was prescribed with topical antibiotic, topical and systemic corticosteroid. Two weeks post-operatively, her graft was melting, oral corticosteroid was stepped up, topical steroid and lubricants were continued and she was under close observation for every 2 weeks. Subsequently, no further melting was noted after 5 months post-operative period (Figure 5(c)).

### 2.6. Case 6

**Figure 6:**
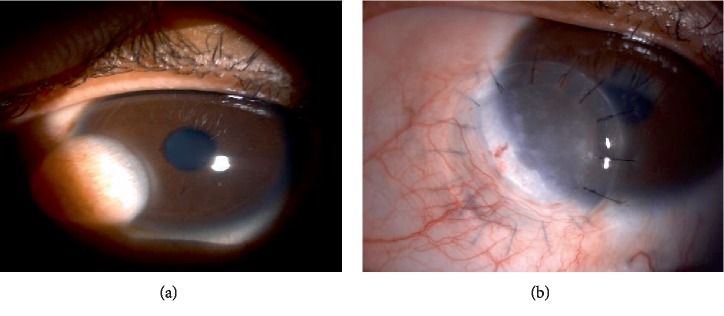
(a) Presence of left eye inferonasal limbal dermoid. (b) Post limbal dermoid removal and corneoscleral graft, 2 weeks post operatively.

A 15-year-old boy with congenital right eye temporal limbal dermoid (Figure 6(a)) was seen in eye clinic. This child has been on regular follow up with refraction. He had amblyopia on right eye. The presence of limbal dermoid on his right eye didn't cross his visual axis.

He underwent excision of the limbal dermoid and corneal patch graft surgery (Figure 6(b)). Post-operatively, the graft looked stable. However his refraction noted to have high astigmatism for which he is being followed up.

## 3. Discussion

Cornea melting is a sequelae of progressive keratolytic process. Untreated cases can lead to cornea perforation. Application of corneal patch graft is another way to address this issue. Corneal patch graft use when the cornea defect is too large until the application of cyanoacrylate tissue adhesive is not recommended (perforation of >1 mm) [5] or when there is cyanoacrylate tissue adhesive application failure [5]. This technique will allow the perseverance of globe integrity, stabilize the eye while waiting for the systemic immunosuppressant effect to take place, clearance of necrotic stroma, which is a source of collagenase and shields the bare stroma from the tears neutrophil [5].

In this series, the decision for patch graft was made due to many reasons. The author attempted corneal patch graft as patients were not responding to pharmacological management.

Small cornea perforation as in case 1, was initially managed with cyanoacrylate glue, however, this method was not fruitful in this case. Application of corneal patch graft helped to seal off the perforation and maintained the anterior chamber. Post-operatively this patient had persistent epithelial defect because of uneven surface. This results in unequal distribution of tear film which can result in persistent epithelial defect. The patient underwent graft re-suturing twice before he responded well. A round trephined corneal patch graft was used in this patient had minimal effect on visual axis [5].

Case 2, 3 and 4 involves the usage of corneal patch graft in Mooren's ulcer. Stepwise approach involving; conjunctival resection, amniotic membrane graft and corneal patch graft with concurrent systemic and topical immunosuppressant, manage to control the disease activity. Conjunctival resection is known to remove the presence of complements, immunoglobulins and inflammatory cells [6]. Two patients underwent conjunctival resection together with cornea patch grafting. These helped to halt cornea melting and protect the graft while waiting to buy time for systemic immunosuppressant to work [5]. For instance, in case 3, following corneal patch graft surgery, the patient endured conjunctiva resection for 3 times before the disease activity managed to be controlled.

Scleral thinning has been shown to respond well with corneal patch graft. As in case 5, the usage of corneal patch graft can be cosmetically acceptable as the curvature of cornea was able to cover large area of scleral bed defect [7]. Conjunctiva flap with subsequent graft opacification can limit the visibility of choroid [7]. Post op management is very crucial in any successful surgery. Tapering down oral corticosteroid too soon may impose the risk of graft melting as in this case, but as soon as the corticosteroid was stepped up, the melting has ceased and the graft remains stable.

Corneal patch graft similarly used in post limbal dermoid excision. It has proven to consume less time and labor force [8]. The cosmetic result is comparable with previous study conducted by Wu et al. Their study using similar method has shown no complication such as tissue over riding, wound dehiscence and suture related complications [8].

All patients who underwent this cornea patch graft surgery were treated with topical corticosteroid and topical antibiotic. The author advocate the usage of topical prednisolone acetate 1% and topical levofloxacin 0.5% along with adequate lubricants. Systemic corticosteroid; oral prednisolone was started peri-operatively to control the ocular inflammatory process. Apart from oral corticosteroid, systemic immunosuppressant such as oral azathioprine was given in cases where the patients were treated for Mooren's ulcer.

## 4. Conclusion

Corneal patch graft has evidence to improve the outcome in treating anterior segment disorders especially involving cornea and sclera. Its advantages have made it possible to produce good post-operative result; be it functionally or cosmetically.
